# Natural Dyes and Their Derivatives Integrated into Organic Solar Cells

**DOI:** 10.3390/ma11122579

**Published:** 2018-12-18

**Authors:** Varun Vohra

**Affiliations:** Department of Engineering Science, University of Electro-Communications, Tokyo 182-8585, Japan; varun.vohra@uec.ac.jp; Tel.: +81-042-443-5359

**Keywords:** organic solar cells, natural dyes, photovoltaic technology, carotenoids, chlorophyll, indigo, multiprotein complex photosystem

## Abstract

Natural photosynthetic systems contain several dyes such as carotenoids or chlorophylls which are adequately arranged to produce efficient photoinduced charge separation and electron transfer. Several research groups have attempted integrating these natural dyes and photosynthetic systems into functional organic solar cells (OSCs) producing power conversion efficiencies (PCEs) up to 0.99%. The studies presented in this short review emphasize that functionalization of natural dyes can considerably improve their PCEs. For instance, chlorophyll derivatives can yield PCEs up to 2.1%, and copolymers produced with isoindigo as an electron-deficient unit generate high PCEs up to 8%, respectively, when combined with fullerene C_70_ based electron acceptors in the OSC active layers. An alternative approach for natural dye integration into OSC architectures is to place these light-harvesting antennas at the interface between the active layer and the charge collection layers in these low-cost photovoltaic devices. This strategy produces large PCE increases up to 35% with respect to OSCs prepared without the interlayer. When light-harvesting systems are combined with silver nanoprisms as interlayers, additional localized surface plasmon resonance effects result in high-performance OSCs that integrate natural photosynthetic systems and demonstrate a PCE over the milestone value of 10%.

## 1. Introduction

**Figure 1:**
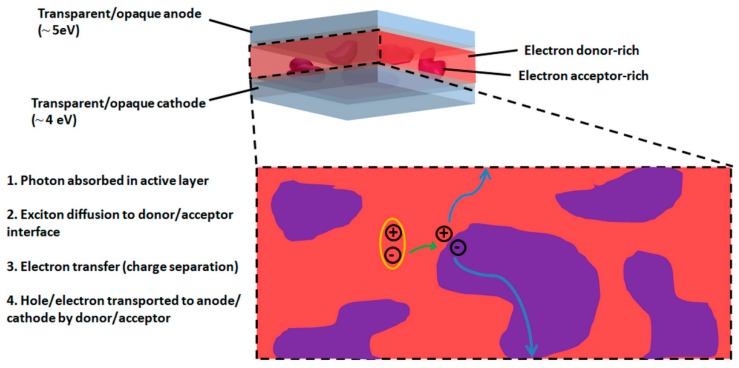
Schematic representation of bulk heterojunction organic solar cells (OSCs). Values in brackets under the electrodes correspond to the typical work functions of anodes and cathodes employed in OSCs.

Organic solar cells (OSCs) have a great potential for the fabrication of low-cost photovoltaic technology which can potentially be integrated into existing infrastructures as semi-transparent photovoltaic windows or into wearable technologies as a flexible power source [1,2,3,4]. Although the OSC active materials initially had a relatively simple molecular structure, engineering the design of low bandgap copolymers through advanced chemistry resulted in a rapid increase in OSC power conversion efficiencies (PCEs) with reported values over 10% since 2015 [5]. More recently, the standard fullerene-based electron acceptors have been replaced with small molecule acceptors in the active layers, thus, further improving the PCE of OSCs over 14% [6]. Unfortunately, the fabrication of these efficient semiconductors requires numerous synthetic steps, thus, generating hazardous organic solvent wastes and considerably increasing the cost of materials for OSC active layers. Natural dyes with adequate optoelectronic properties which can be extracted at an extremely low-cost from vegetables, fruits or plants consequently provide a more sustainable alternative to the conventional OSC active layer synthetic materials. In fact, natural dyes have been extensively studied for their use in dye-sensitized solar cells (DSSCs) [7]. However, transferring this knowledge to OSCs can be quite challenging as the working principles and architectures of these third-generation photovoltaic devices are quite different from each other. In DSSCs, the light-harvesting dye is attached to metal oxide semiconductors (typically TiO_2_ or ZnO nanoparticles), and once charge separation occurs, the hole is transferred from the organic dye to an electrolyte which carries out the function of hole transport to the anode while electrons percolate through the inorganic nanoparticles to the cathode. On the other hand, in OSCs, the electron donor and the electron acceptor carry out light harvesting and charge transport functions, making charge mobility in the organic semiconductors a critical factor to ensure efficient OSCs operation (Figure 1). In a recent review, Ravi et al. emphasized the differences between natural light-harvesting systems and bio-inspired OSCs by considering several device architecture designs and correlating the working mechanisms of natural photosynthesis and OSCs with the energetic diagrams of the various active molecules [8].

**Figure 2:**
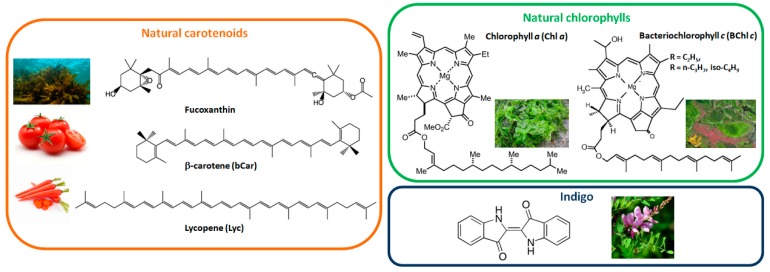
Molecular structures of natural dyes reviewed in this study along with the images of the typical plants, fruits or bacteria they can be extracted from.

Studies on natural dyes employed in Schottky diodes type OSCs can be found in the scientific literature since the late 20th century [9,10,11,12,13]. Particular attention was given to natural pigments such as carotenoids, chlorophylls, indigo or photosystem I (PSI) and photosystem II (PSII) that contain carotenoid and chlorophyll pigments [14]. However, these OSCs are based on a single semiconductor in their active layer, and, thus, the amount of photogenerated charges remains relatively low. As OSCs evolved into more advanced device architectures such as bulk heterojunction (BHJ) solar cells (Figure 1), a rapid increase in the PCE of OSCs fabricated with natural dyes and their derivatives could be observed. BHJ solar cells are based on electron donor and electron acceptor materials which are co-deposited by thermal evaporation or solution-process to form active layers with donor-rich and acceptor-rich domains. Active layers can also be produced through a sequential deposition of electron donor and electron acceptor materials. When a photon is absorbed by the donor, the formed exciton diffuses to a donor/acceptor interface where an electron from the lowest unoccupied molecular orbital (LUMO) of the donor is transferred to the LUMO of the acceptor, thus, generating electron-hole pairs. Similarly, when the photon is absorbed by the acceptor, once the exciton reaches a donor/acceptor interface, an electron is transferred from the highest occupied molecular orbital (HOMO) of the donor to the HOMO of the acceptor. To ensure that charges are generated efficiently, the continuous donor or acceptor domains should be smaller than the exciton diffusion length which is typically in the order of a few tenths of nm. Once the charges are generated, holes and electrons will percolate through donor and acceptor phases to the anode and cathode, respectively. In this review, I will discuss previous studies in which natural dyes were successfully integrated into bilayer or BHJ–OSCs in their natural (as extracted) or functionalized forms, as well as when employed as building blocks for the synthesis of complex molecular structures. Although several attempts have been made to fabricate photovoltaic devices using natural dyes directly extracted from exotic fruits [15,16], in this review, I will focus on molecules based on carotenoids, chlorophylls, indigo, and natural photosynthetic or light-harvesting systems containing carotenoids and chlorophylls such as PSI, PSII or LHCII (Figure 2).

The first section of this review will focus on successful examples of as extracted natural dyes or photosynthetic systems employed as the principal active material in OSCs. After that, I will review several studies related to chlorophyll and indigo derivatives which emphasize that chemical modification of natural dyes may be an adequate strategy when aiming for high-performance bio-OSC fabrication. Finally, in the last section of this review, an alternating strategy, namely, natural products as light-harvesting interlayers will be presented which demonstrates that energy transfer from natural systems can be efficiently employed to improve the short-circuit current density (*J*_sc_) of OSCs based on conjugated polymer electron donors. As the device performances of each successful case will be presented here, this review will give the opportunity for researchers in biophotovoltaics to select the adequate materials and strategies to fulfill their objectives which can either be the fabrication of OSCs using products extracted from biomass at an extremely low cost or developing new materials that integrate natural dyes to produce high performance devices.

## 2. As Extracted Natural Dyes in OSC Active Layers

**Figure 3:**
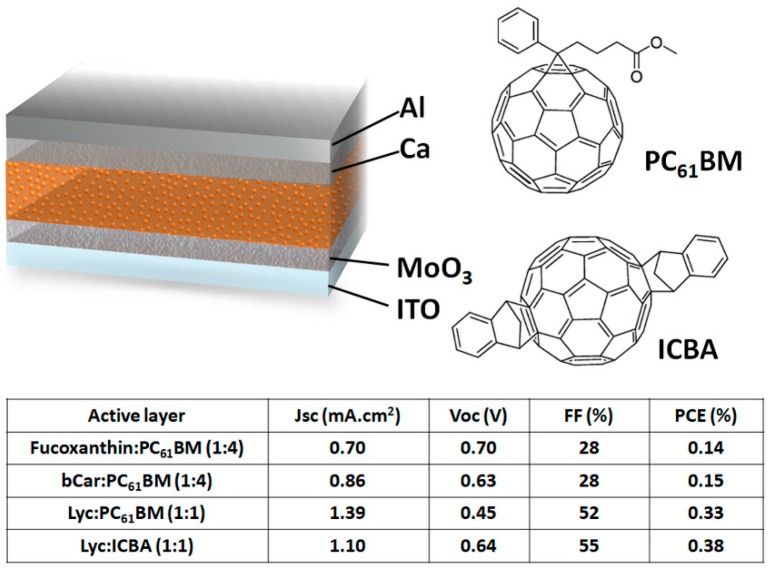
Schematic representation of the device architecture, molecular structures of electron acceptors and photovoltaic performances of the devices prepared in Reference [20].

Carotenoids (Figure 2) have a molecular structure that is very similar to polyacetylene oligomers. β-carotene (bCar) is one of the pigments found in PSI and PSII [14], which also gives tomatoes or red carrots their color [17]. Lycopene (Lyc) can be extracted from tomatoes using a sustainable solvent such as limonene [18]. Fucoxanthin is mostly found in brown algae and extracted with organic solvents such as dimethyl ether [19]. These three natural carotenoids were studied by Wang et al. in combination with two well-known C_60_ fullerene derivatives, namely, PC_61_BM and ICBA (Figure 3) [20]. As expected from their similarities with polyacetylene, the three carotenoids exhibited a p-type organic semiconductor behavior with hole mobilities of 0.81 × 10^−4^ cm^2^/(Vs), 0.18 × 10^−4^ cm^2^, and 210 × 10^−4^ cm^2^ measured for fucoxanthin, bCar, and Lyc, respectively. Due to the low hole mobilities of fucoxanthin and bCar, low donor:acceptor ratios (1:4) were necessary to produce relatively efficient fucoxanthin:PC_61_BM and bCar:PC_61_BM BHJ–OSCs. On the other hand, as Lyc demonstrated a hole mobility three orders of magnitude higher than the other two carotenoids, a 1:1 donor:acceptor ratio was used in the Lyc:PC_61_BM devices. Fucoxanthin has a shorter conjugation length than bCar, which is also shorter than that of Lyc. Therefore, different values of HOMO levels were found for the three carotenoids with values of −5.6 eV, −5.1 eV, and −4.8 eV, respectively for fucoxanthin, bCar, and Lyc. The photovoltaic performances of the regular architecture carotenoids:fullerene derivatives BHJ–OSCs are presented in Figure 3. As expected, a donor with a low-lying HOMO, such as fucoxanthin, yields high *V*_oc_ values of 0.70 V when combined with PC_61_BM thanks to the enlarged gap between the LUMO of the acceptor (−3.9 eV) and the HOMO of the donor. The higher HOMO levels of bCar and Lyc result in a gradual decrease of the *V*_oc_ value in carotenoid:PC_61_BM devices to 0.63 V and 0.45 V, respectively. By replacing PC_61_BM with ICBA in Lyc-based OSCs, the higher LUMO of ICBA produced a *V*_oc_ of 0.64 V. Although the *V*_oc_ of fucoxanthin and bCar devices had similar values to those prepared with synthetic electron donors such as P3HT [21], their low *J*_sc_ and fill factors (FFs) resulted in PCEs around 0.15%. On the other hand, both Lyc:PC_61_BM and Lyc:ICBA yielded FFs over 50% and *J*_sc_ over 1 mA/cm^2^, thus, producing PCEs of 0.33% and 0.38%, respectively. To my knowledge, these are the highest reported efficiencies for natural carotenoid BHJ–OSCs up to now. In a follow-up study, the authors also attempted to combine Lyc with derivatives of the product of chlorophyll breakdown [22]. However, this attempt to mimic natural photosynthesis resulted in low-performance devices with maximum PCE values of 0.045%. 

**Figure 4:**
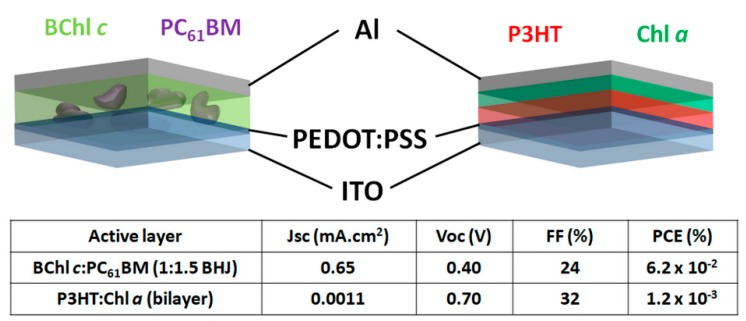
Schematic representations of the device architectures and photovoltaic performances of the devices prepared in References [23,24].

Previous studies suggest that when ordered in an adequate manner, chlorophylls exhibit ambipolar properties [23]. This implies that they can be used as both electron donor and electron acceptor in BHJ–OSCs. In fact, bacteriochlorophyll *c* (BChl*c*, extracted from *Chlorobiumtepidum*) and chlorophyll *a* (Chl*a*, extracted from algae) have been employed as electron acceptor and electron donor combined in regular architecture OSC active layers with P3HT and PC_61_BM, respectively [23,24]. The optimized photovoltaic performances of these OSCs are presented in Figure 4.

**Figure 5:**
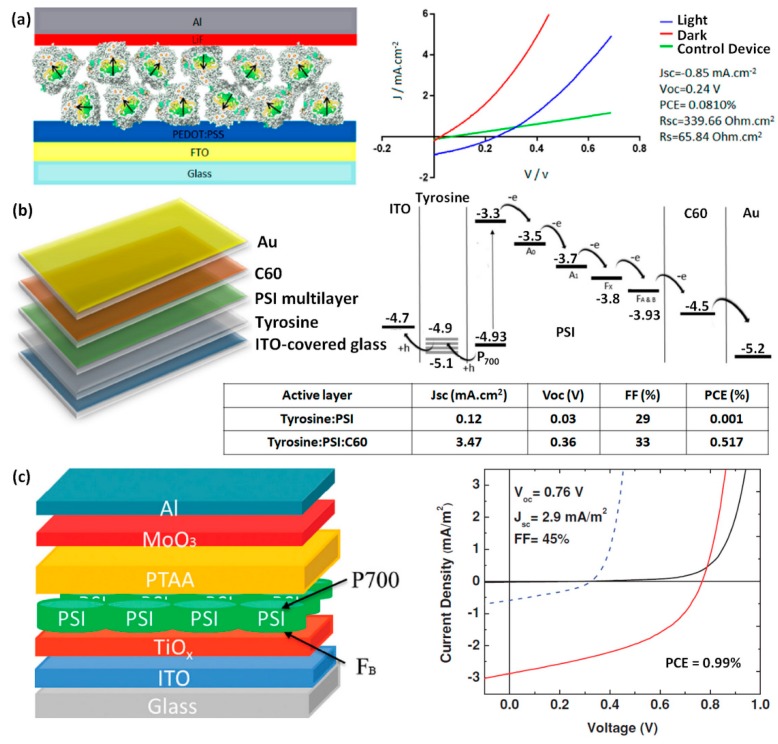
Schematic representations of device architectures and photovoltaic properties of the PSI-based OSCs fabricated in (**a**) Reference [28], (**b**) Reference [29], and (**c**) Reference [32]. Reprinted with permission from References [28,29,32]. Copyright 2017 American Chemical Society. Copyright 2017 Elsevier. Copyright 2014 John Wiley and Sons.

Although Lyc and BChls have similar HOMO levels around –4.8 eV [20,25], OSCs employing Lyc and BChl*c* as electron donors with PC_61_BM as acceptor produced PCEs of 0.33% and 0.062%, respectively. The five times higher PCE in Lyc:PC_61_BM OSCs resulted from approximately two times larger *J*_sc_ and FF compared to BChl*c*:PC_61_BM devices, in addition to their slightly higher *V*_oc_. We should note here that the presence of Ca between the active layer and the Al in the carotenoid devices promoted efficient electron collection which may explain the differences seen in the two devices. Other factors such as disordered BChl*c* molecules in the active layer or lower intrinsic hole mobilities may have also caused decreases in *J*_sc_, *V*_oc_, and FF in the BChl*c* devices compared to the Lyc ones. Nevertheless, the BChl*c*:PC_61_BM devices clearly indicated that BChls can be employed in working OSCs as electron donor. Surprisingly, the devices employing P3HT:Chl*a* BHJ active layers did not display any photovoltaic effect [24]. On the other hand, sequential deposition of P3HT and Chl*a* into bilayer active layers produced a small photovoltaic effect and PCEs of 0.0012% when a 20 mW/cm^2^ AM1.5 illumination was used. As P3HT and Chl*a* have similar HOMO levels around −4.7 eV, considerable charge recombination probably occurs in these devices, which could explain the higher performance observed at lower illumination intensity. Assuming that efficient charge separation occurs at the donor/acceptor interface, such behavior also implies that electron mobility in the Chl*a* deposited on top of P3HT is fairly low. In fact, Mustain et al. measured the electron mobility to vary between 1× 10^−4^ cm^2^/(Vs) and 4× 10^−4^ cm^2^/(Vs) in disordered spin-coated Chl*a* thin films [26]. Natural photosynthesis teaches us that well-arranged combinations of natural dyes can produce efficient charge transfer and charge transport [27]. As carotenoids and Chls are already organized in PSI, Kazemzadeh et al. recently attempted to use the whole photosystem extracted from spinach (PSI) as the only active material in regular device architectures (Figure 5a) [28]. These bio-inspired OSCs produced a PCE of 0.08% with a *J*_sc_ of 0.85 mA/cm^2^, a relatively low *V*_oc_ of 0.24 V, and an FF of approximately 40%. The surface roughness of the deposited PSI films (root mean square value of 16.12 nm) largely exceeded the thickness of lithium fluoride (LiF), suggesting that PSI could yield higher performances in adequate device architectures. In fact, the same group published a second study in which they employed PSI in OSCs with PCEs reaching 0.52% (Figure 5b) [29]. 

In the second device architecture they proposed, PEDOT:PSS was replaced by Tyrosine as the hole transporting layer and the PSI active layer was covered with a C_60_ layer from a water-dispersed solution and the devices exhibited a *J*_sc_ of 3.47 mA/cm^2^. The relatively low FF value (33%) suggests that further improvements in the device design could lead to enhanced PCEs of the devices. As displayed in Figure 5b, the large energy gap between the LUMO of C_60_ and the work function of Au may result in inefficient electron collection. Nonetheless, the devices fabricated with Tyrosine and C_60_ largely overcame initial attempts at fabricating solid-state photovoltaic devices using PSI and reaction centers extracted from spinach as active materials [30]. Recent advances indicate that inverted OSC architectures with metal oxide electron transport layers have a tendency to yield higher performances than their conventional architecture equivalents [5,31]. The work by Gordiichuk et al. confirmed that the design of OSC architecture integrating natural products plays an essential role in obtaining high PCEs (Figure 5c) [32]. In their inverted OSCs based on PSI active layers, efficient charge collection at the PSI/TiO*_x_*, and the PSI/PTAA interfaces produced an FF of 45% and a high *V*_oc_ of 0.76 V. Despite a slight decrease in *J*_sc_ (2.9 mA/cm^2^) with respect to the regular device architectures with Tyrosine and C_60_, a PCE close to 1% was obtained in these inverted architecture PSI OSCs. This is the highest PCE value reported for unmodified natural product active materials in OSCs. As we will see in the following section, functionalization of natural dyes or their use as building blocks for electronic material design can further improve the performances of OSCs and/or facilitate the formation of adequate morphologies in biological system-inspired active layers. 

## 3. Efficient OSCs Employing Natural Dye Derivatives or Synthetic Equivalents as Active Materials

**Figure 6:**
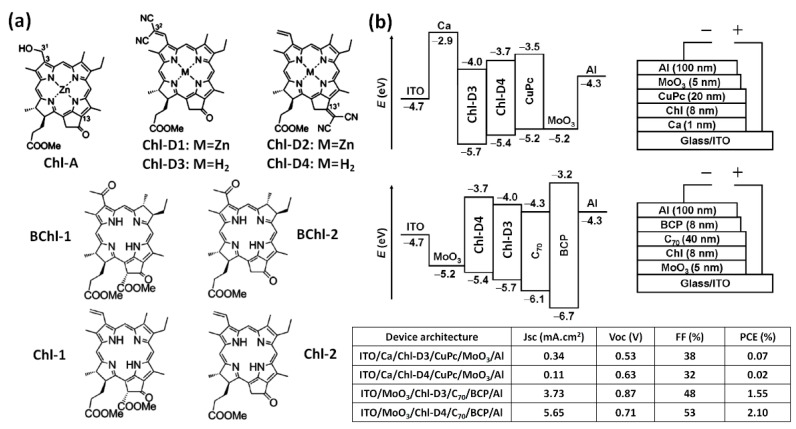
(**a**) Molecular structures of chlorophyll-derivatives; (**b**) device architecture and performances of chlorophyll-derivatives as either electron donor or electron acceptor in OSC active layers. Reprinted with permission from References [33,34,35]. Copyright 2013 Elsevier. Copyright 2017 Elsevier. Copyright 2018 American Chemical Society.

**Figure 7:**
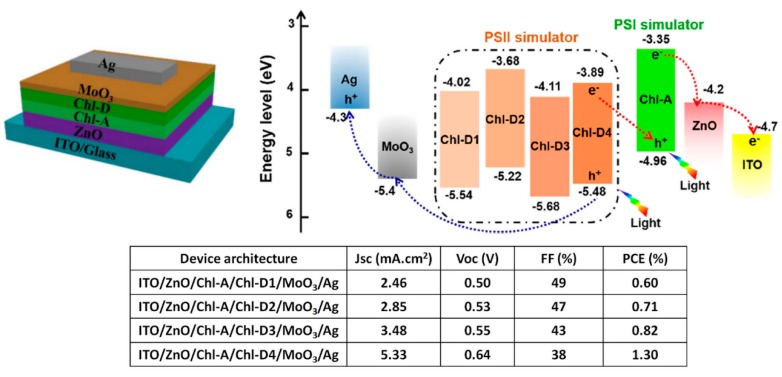
Schematic representation of device architecture and energy levels along with the device performances of all chlorophyll-derivatives active layer OSCs. Reprinted with permission from Reference [35]. Copyright 2018 American Chemical Society.

Japanese researchers have developed several approaches to prepare substituted derivatives of Chls and BChls to explore their potential use as the active material in OSCs [22,33,34,35]. Although these systems are not natural, their results provide very interesting insight on the use of (B)Chl derivatives in OSC active layers. For instance, Wang et al. prepared two metal-free dicyano-functionalized Chl derivatives (Chl-D3 and Chl-D4 in Figure 6a) which exhibit ambipolar charge transport properties [33]. When employed as electron acceptors with Lyc [22], or Copper phthalocyanine (CuPc) [33], Chl-D3 and Chl-D4 exhibited low photovoltaic performances in OSCs with PCEs below 0.1% (Figure 6b). Even though their electron mobilities are higher than their hole mobilities, efficient bilayer OSCs with 8 nm-thick Chl-D3 or Chl-D4 electron donors topped with a 40 nm-thick C_70_ layer were produced. The low-lying HOMO of Chl-D3 (−5.7 eV) yielded a high *V*_oc_ of 0.87 V. However, the large energy gap between the HOMO and the work function of MoO_3_ (−5.2 eV) reduced the hole collection efficiency, thus, producing a *J*_sc_ and an FF below 5 mA/cm^2^ and 50%, respectively. As the HOMO of Chl-D4 is 0.3 eV higher than that Chl-D3, a notable increase of *J*_sc_ and FF was obtained in Chl-D4:C_70_ OSCs with only a small drop of the *V*_oc_. Note that the two different positions of the dicyano-functionalized carbon in these Chl derivatives also resulted in slight modifications in their absorption spectra, which may contribute to the *J*_sc_ enhancement observed in Chl-D4 OSCs. Chl-D4:C_70_ OSCs demonstrated a *J*_sc_ of 5.65 mA/cm^2^, a *V*_oc_ of 0.71 V, and an FF of 53%, thus, producing a PCE of 2.1% and clearly demonstrating that Chl derivatives can act as efficient electron donors in thin bilayer OSCs. The authors developed additional BChl and Chl derivatives (BChl-1, BChl-2, Chl-1, and Chl-2 in Figure 6a) but these (B)Chl:C_70_ OSCs could not overcome the results obtained with Chl-D4 [34]. Although their initial study suggested that Chl derivatives cannot be used as efficient electron acceptors in OSCs, Duan et al. recently fabricated inverted device architectures (ITO/ZnO/active layer/MoO_3_/Ag) in which both the electron donor and electron acceptor were Chl derivatives. Unlike their previous attempts with metal-free Chl-derivatives, the electron acceptor in these devices was a Zn-based chelate (Chl-A). Note that the common metal found in natural Chls is Mg. The HOMO and LUMO levels of Chl-A were −4.96 eV and −3.35 eV, respectively. Both these values were higher than the HOMO/LUMO levels of the electron donors (Chl-D1, Chl-D2, Chl-D3, and Chl-D4) in the inverted OSCs (Figure 7). The authors proposed a non-conventional working mechanism for these OSCs which mimicked PSII/PSI interactions in natural photosynthetic systems. In their proposed mechanism, the exciton was first formed on the PSII simulator (Chl-A), and the charge separation occurred at the Chl-A/ZnO interface through an electron transfer from the LUMO of Chl-A to ZnO. The following step involved a second exciton formed on the PSI simulator (electron donor, Chl-Ds) with the electron promoted to the LUMO of Chl-Ds through absorption and transferred to the HOMO of Chl-A. Although this peculiar working mechanism would explain the high FFs obtained despite the higher HOMO level of the acceptor with respect to those of the donors, clear evidence (e.g., using time-resolved spectroscopy or photoluminescence quenching measurements) of such two-step operation is lacking in their study. Nonetheless, the Chl-A/Chl-Ds bilayers yield PCEs above 0.5%, and it is, thus, safe to say that these devices do not operate following a conventional OSC working principle. 

**Figure 8:**
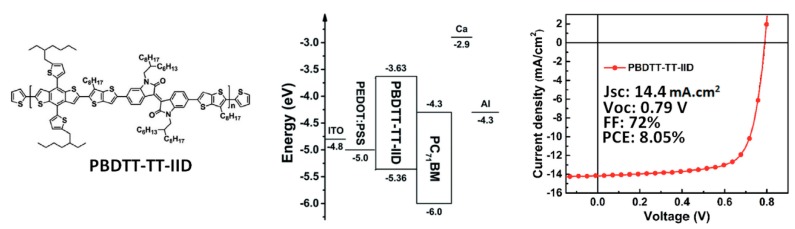
Molecular structure of PBDTT-TT-IID, energy diagram and photovoltaic properties of PBDTT-TT-IID-based OSCs. Reprinted with permission from Reference [49]. Copyright 2016 Royal Society of Chemistry.

In summary, the use of functionalized Chls or more precisely, synthetic Chl-like structures can produce OSCs with PCEs up to 2.1%. The necessity for chemical modification of natural molecules or synthetic routes required for Chl equivalent production diminishes the advantage of using natural products in OSCs. In fact, the chemical modification of natural molecules is not limited to Chls and can also facilitate the process of indigo-based OSCs. Indigo is a natural dye that has been extensively employed as a pigment for the textile industry and, as demonstrated in a review by Głowacki et al., indigo-based molecules exhibit a great potential as a building block for organic electronic materials, including active materials for OSCs [36]. Similarly to Chls, indigo exhibits ambipolar properties when employed in organic electronic devices [37]. Deposition of indigo by solution-process can be relatively challenging due to its low solubility in organic solvents in its natural form. Głowacki et al. presented a simple method for solution-processed indigo thin films deposition by functionalizing the natural dyes with *tert*-butoxy carbonyl groups which can be removed by annealing the deposited films at 200 °C [38]. The functionalized indigo dyes were also blended with P3HT into BHJ active layers, but the high-temperature annealing necessary to recover the natural indigo molecular structure resulted in micrometer-scale phase separated domains, leading to a *J*_sc_ below 0.5 mA/cm^2^. Judging from the *J–V* curve of their OSC, a *V*_oc_ of approximately 0.35 V was achieved, and the PCE still remained below 0.1%. However, the study confirms the n-type character of indigos. In fact, the electron deficient isoindigo has been extensively used as a building block to design small molecular and polymeric semiconductor materials for OSC active layers [39,40,41,42,43,44,45,46,47,48,49]. For instance, a polyisoindigo synthesized by simple coupling was blended in a 1:1 ratio with P3HT to produce OSCs with a *J*_sc_ of 1.91 mA/cm^2^, a *V*_oc_ of 0.62V and an FF of 41%, resulting in a PCE of 0.47% [39]. Electron-deficient building blocks can be combined with electron donor units to engineer the frontier orbitals energy levels and optical properties of small molecules and low bandgap copolymers, which has proven to be an efficient strategy to enhance the performances of OSCs [1,2,5,6,50]. Following this approach, a few research groups have put a great effort into the design of isoindigo-based materials attached to electron donors such as thiophene groups. Mei et al. synthesized a small molecule with a central isoindigo unit bonded to a bithiophene on each side in a donor–acceptor–donor (D-A-D) configuration [40]. When combined with PC_61_BM in BHJ–OSC active layers, the D-A-D molecules generated a *J*_sc_ of 6.3 mA/cm^2^, a *V*_oc_ of 0.74 V, and an FF of 38%, resulting in a PCE of 1.76%. On the other hand, isoindigo or substituted indigos have been associated with various well-known electron donating units including anthracene, naphthalene, and thiophenes to synthesize electron donors for OSCs that produce PCEs below 5% [41,42,43,44,45]. To generate PCEs over 5%, further optimization of the isoindigo-based materials was performed through the addition of alkyl substituents and by associating them with well-engineered D units such as terthiophenes (PCE: 6.3%) [46], or benzodithiophenes and thiophenylbenzodithiophenes linked to isoindigos through bithiophene (PCE: 7.31%) [47], or through thienothiophenes (max PCE: 8.05%) [48,49]. The best performing material (PBDTT-TT-IID in Figure 8) produced PCEs on par with synthetic materials such as PTB7 which is among the highest performing polymer semiconductors [51]. 

The BHJ–OSCs prepared with PBDTT-TT-IID as the electron donor and PC_71_BM as the electron acceptor exhibited a *J*_sc_ of 14.4 mA/cm^2^, a *V*_oc_ of 0.79 V, and an FF of 72%, thus, producing a PCE of 8.05%. These promising results clearly indicate that isoindigos have a great potential for the synthesis of well-performing donor materials for BHJ–OSCs. However, as this strategy relies on advanced chemistry to engineer the molecular structure of the conjugated polymer and, in particular, the PBDTT-TT unit, the low-cost aspect of natural conjugated molecules is somewhat shadowed. It is, therefore, important to evaluate whether a different strategy using as extracted molecules or those requiring minimal chemical modifications can yield similar results to those obtained in PBDTT-TT:PC_71_BM BHJ–OSCs.

## 4. OSCs with Natural Complexes as Light-Harvesting Interlayers 

**Figure 9:**
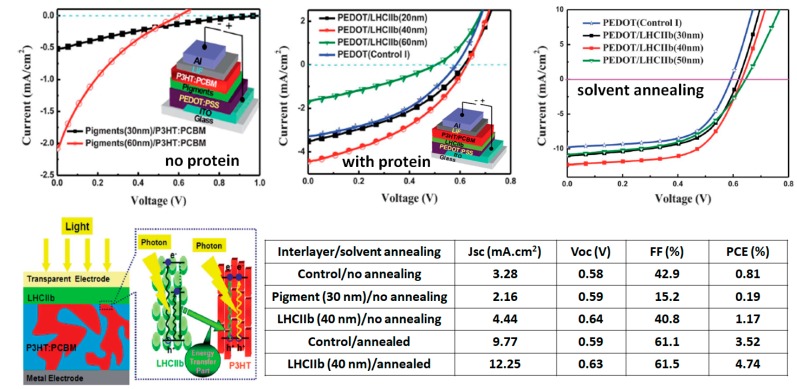
Comparative *J*–*V* characteristics of P3HT:PC_61_BM OSCs with pigments or LHCIIb as interlayer material. Schematic representation of the working principle and photovoltaic performances. Adapted with permission from Reference [54]. Copyright 2012 Royal Society of Chemistry.

Ternary OSCs have been receiving growing interest in recent years as they produce large enhancements in device performance through a broader light-harvesting capacity [52]. Unlike binary solar cells, the working principle of ternary solar cells sometimes relies on resonant energy transfer mechanisms from a light-harvesting antenna to materials with high charge transport properties [53]. Most ternary OSC active layers are produced by co-deposition of the three active materials, but sequential deposition of a light-harvesting antenna and a binary BHJ active layer also constitutes a viable strategy to improve the OSC device performances. This approach was explored with natural dyes and light-harvesting complexes integrated as interlayers in BHJ–OSCs [32,51,54,55]. For example, Yao et al. investigated the LHCIIb light-harvesting complex inclusion into P3HT:PC_61_ BMBHJ–OSCs (Figure 9) [54]. In as deposited (unannealed) devices, when dyes extracted from the LHCIIb (a mixture of carotenoids and Chls) were employed without the surrounding protein as interlayer material, the device performances were considerably reduced. The *J*–*V* characteristics of these devices displayed an S-shape, which is typically assigned to high series and shunt resistances in the device. On the other hand, insertion of a 40 nm-thick LHCIIb layer between PEDOT:PSS and the BHJ active layer produced a notable enhancement in PCE as the *J*_sc_ increased from 3.28 mA/cm^2^ to 4.44 mA/cm^2^ for the OSCs without and with LHCIIb interlayers, respectively. 

A similar *J*_sc_ enhancement was seen in “solvent annealed” P3HT:PC_61_BM OSCs. Note that the solvent annealing here refers to an increase in drying time with a higher boiling point solvent. The spectral response of the photogenerated current in devices with and without the LHCIIb interlayers indicates that the *J*_sc_ enhancement results from a resonant energy transfer from LHCIIb to P3HT. The OSCs fabricated with LHCIIb interlayers produced a PCE 35% higher than those without the interlayer, with a maximum PCE of 4.74%. The insertion of light-harvesting interlayers is a well-known strategy in OSCs, but the performance enhancements presented here largely overcome those obtained using additional conjugated polymer or fullerene interlayers [56,57]. Liu et al. applied a similar strategy with LHCII (extracted from peas) inserted between ZnO nanoparticles and PTB7:PC_71_BM or PTB7-Th:PC_71_BM active layers in inverted OSC architectures [51]. The insertion of the natural photosynthetic system at the ZnO nanoparticle/active layer interface produced a 10% and 15% increase in PCE for PTB7:PC_71_BM OSCs and PTB7-Th:PC_71_BM OSCs, respectively. Unlike the study from Yao et al., in which PCE increase was related solely to *J*_sc_ improvement, the PCE enhancements here resulted from combined improvements in *J*_sc_ and FF which were attributed to improved interfacial properties. These studies suggest that LHCII(b) insertion could be a simple and cost-effective method to enhance the properties of regular and inverted OSCs. 

**Figure 10:**
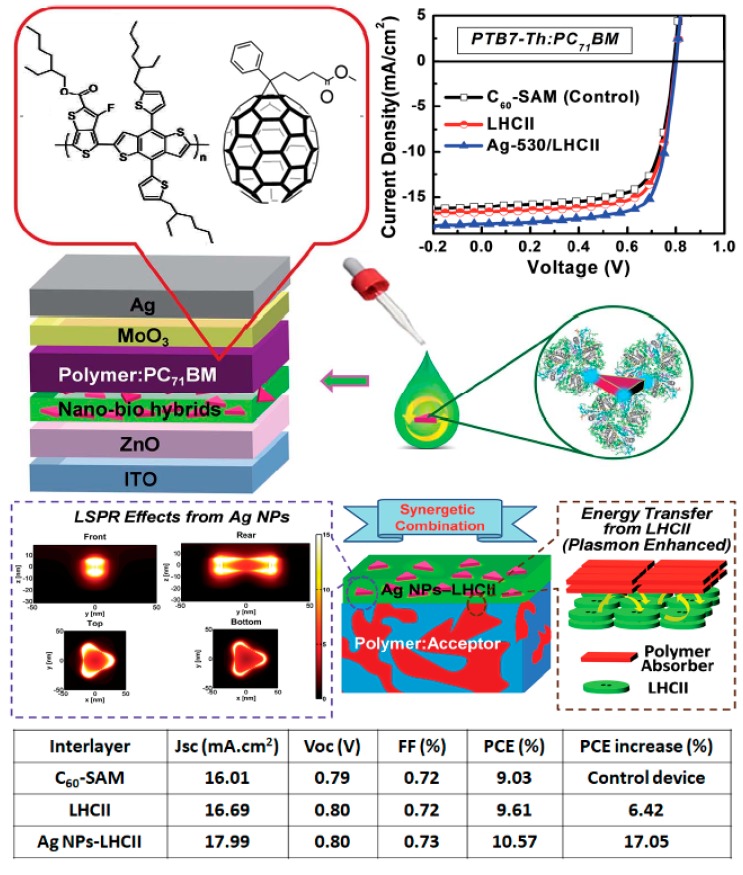
*J*–*V* characteristics, schematic representations of the device architecture and working principle as well as device performances of PTB7-Th:PC_71_BM OSCs employing Ag NPs-LHCII based nano-bio hybrids as interlayer material. Schematic representation of the working principle and photovoltaic performances. Reprinted with permission from Reference [55]. Copyright 2016 Royal Society of Chemistry.

In fact, in 2016, Yao et al. demonstrated equivalent enhancements when using more homogeneous ZnO layers prepared by a sol–gel approach rather than ZnO nanoparticles [55]. Although the best results were obtained with PTB7-Th:PC_71_BM active layers, a performance enhancement of around 6% (control device: ITO/ZnO/C_60_-self-assembled monolayer; C_60_-SAM/active layer/MoO_3_/Ag) was observed for the three active layers they studied. The authors pushed the interlayer strategy one step further by combining LCHII and silver nanoprisms (Ag NPs) into plasmonic light-harvesting nano-bio hybrid interlayers for OSCs (Figure 10). The Ag NPs produced a localized surface plasmon resonance (LSPR) effect which enhances the LCHII light absorption properties. As LCHII acts as antenna material in these OSCs, increasing the absorption from LCHII provides more energy to the polymer absorbers through energy transfer. As a result, the *J*_sc_ was considerably enhanced from 16.01 mA/cm^2^ for control devices to 17.99 mA/cm^2^ for OSCs fabricated with the nano-bio hybrid interlayers. As the other photovoltaic parameters (*V*_oc_ and FF) remain constant upon insertion of the interlayer, the large *J*_sc_ enhancement was translated into a PCE increase of 17% for the Ag NPs-LHCII devices with respect to the control OSCs fabricated with C_60_-SAM. The average PCE increased from 9.03% (control devices) to 9.61%, and 10.57% for OSCs fabricated with LHCII and Ag NPs-LHCII interlayers, respectively. A maximum value of 10.88% was measured for the nano-bio hybrid interlayer devices, making them one of the top performing OSCs based on fullerene derivatives as electron acceptors. We should emphasize here that plasmonic structures can readily enhance the performances of OSC devices without the necessity for the LHCII. For instance, Oh et al. demonstrated that the insertion of metal nanodots can produce over 34% of PCE enhancement in PTB7:PCBM OSCs [58]. Although we could argue that the insertion of Ag NPs adds to the fabrication cost of OSCs, these results demonstrate that natural products can be efficiently engineered to improve the performances of BHJ–OSCs.

## 5. Conclusions

A summary of typical device performances achieved with the various strategies presented in this review is summarized in Table 1. When unmodified natural dyes, such as carotenoids or chlorophylls, are employed as an electron donor or electron acceptor in OSC active layers, the resulting cost-effective OSCs exhibit fairly low performances with PCEs generally below 0.5%. In PSI, carotenoids and chlorophylls are naturally organized to promote efficient electron transfer and, thus, PSI OSCs produce PCEs close to 1%, the best performances obtained with as extracted natural active materials. However, molecular engineering of chlorophyll-derivatives and indigo-based materials can considerably increase the performances of bio-OSCs. OSCs with active layers entirely composed of chlorophyll-derivatives produce PCEs up to 1.3%. When the chlorophyll-derivatives are used as electron donor combined with C_70_ as the acceptor, the resulting OSC PCEs can be enhanced up to 2.1%. The electron deficient isoindigo can be employed as a building unit for alternating donor–acceptor type low bandgap copolymers with PCEs over 8% in BHJ–OSCs. Although these materials exhibit high photovoltaic performances, the extensive chemical modifications render the low-cost aspect of the natural dyes irrelevant. Several research groups have proposed an alternative approach based on natural photosynthetic light-harvesting systems such as LHCII to improve the performances of OSCs. Light-harvesting systems have been inserted between the active layer and charge transporting layers as antenna materials in the well-known P3HT:PC_61_BM OSCs as well as other polymer:fullerene OSCs. A remarkable 35% increase in PCE was obtained in P3HT:PC_61_BM devices but when used in other polymer:fullerene systems such as PTB7:PC_71_BM or PTB7-Th:PC_71_BM, the resulting PCE improvement was much lower. By combining the antenna effect with LSPR effects using Ag NPs, a large enhancement in the *J*_sc_ of PTB7-Th:PC_71_BM devices can be observed producing PCEs over the milestone value of 10%. 

In other words, the methods to include natural dyes or their derivatives into OSC architectures each have several advantages and drawbacks. When natural dyes are used as extracted as electron donors in OSC active layers, functional devices can be fabricated, but they only yield fairly low PCEs with values below 1%. It will consequently be very interesting to observe how these semiconductors behave when they are associated with newly developed non-fullerene derivatives such as ITIC [2,6]. Natural photosynthetic systems as interlayer material do not hinder the charge collection in OSCs and provide the means to improve the device *J*_sc_ by harvesting a larger amount of sunlight. The large performance improvement achieved with photosynthetic systems as interlayers also demonstrates that the concept of ternary active layers employing natural antenna systems is a research direction worth pursuing. Although large molecular complexes such as PSI or LHCII would be difficult to efficiently blend into ternary BHJ active layers, the inclusion of smaller dyes (carotenoids, chlorophylls, indigos) may provide an alternative path to large *J*_sc_ enhancements in OSCs without the necessity for additional advanced chemistry. The most promising active materials based on natural pigments remain those that have undergone extensive chemical modifications with particularly high performances achieved when isoindigos are used as building blocks for low bandgap copolymers synthesis. However, the interlayer and chemical modification strategies have the major drawback of further increasing the cost of OSCs. The field of bio-OSCs is still fairly recent and I hope that it will continue to grow in the upcoming future. In particular, I believe that by further engineering the deposition process and active layer morphology of natural dye (carotenoids or chlorophylls) OSCs, low-cost OSCs with PCEs over 1% are well within reach. Although the photovoltaic performances of such bio-OSCs would still pale in comparison to OSC based on synthetic materials, their cost-performance index would be much higher than OSCs employing well-engineered synthetic materials. The short and long-term stability of OSCs employing natural dyes and their derivatives should also be studied as this will be a key factor in assessing their real potential for commercial applications.

## Figures and Tables

**Table 1:** Typical functional devices performances with the various strategies based on natural dye or natural dye derivative presented in this review.

“Natural” Material	Chemical Modifications	Function	Bottom Electrode; Top Electrode	*J*_sc_ (mA/cm^2^)	*V*_oc_ (V)	FF (%)	PCE (%)
Carotenoids [20]	Natural (Lyc)	Electron donor	ITO/MoO_3_; Ca/Al	1.1	0.64	55	0.38
Carotenoids and Chl [22]	Natural (Lyc) and synthetic equivalent (Chl-D4)	Electron donor; electron acceptor	ITO/MoO_3_; Ca/Al	0.23	0.85	23	0.05
Chls [24]	Natural (Chl*a*)	Electron acceptor	ITO/PEDOT:PSS; Al	0.001	0.70	32	0.001
Chls [23]	Natural (BChl*c*)	Electron donor	ITO/PEDOT:PSS; Al	0.65	0.40	24	0.06
Chl-D3 [33]	Synthetic equivalent of chlorophyll derivative	Electron acceptor	ITO/Ca; MoO_3_/Al	0.34	0.53	38	0.07
Chl-D4 [33]	Synthetic equivalent of chlorophyll derivative	Electron donor	ITO/MoO_3_; BCP/Al	5.65	0.71	53	2.10
Chl-A and Chl-D4 [35]	Synthetic equivalent of chlorophyll derivatives	Electron donor; electron acceptor	ITO/ZnO; MoO_3_/Ag	5.33	0.64	38	1.30
PSI [29]	Natural	Active layer	ITO/Tyrosine; C_60_/Au	3.47	0.36	33	0.52
PSI [32]	Natural	Active layer	ITO/TiO*_x_*; PTAA/MoO_3_/Al	2.9	0.76	45	0.99
Indigo [38]	Derivative	Electron acceptor	ITO/PEIE; MoO*_x_*/Ag	0.2~0.5	~0.35	-	<0.1
Isoindigo [39]	Homopolymer	Electron acceptor	ITO/PEDOT:PSS; LiF/Al	1.91	0.62	41	0.47
Isoindigo [49]	Building block for p-type copolymer	Electron donor	ITO/PEDOT:PSS; Ca/Al	14.4	0.79	72	8.05
LHCIIb [54]	Natural	Antenna interlayer	ITO/PEDOT:PSS; LiF/Al	12.3	0.63	62	4.74
LHCII [55]	Natural	Antenna interlayer	ITO/ZnO; MoO_3_/Ag	16.7	0.80	72	9.6
LHCII [55]	Modified with Ag NPs	Plasmonic interlayer	ITO/ZnO; MoO_3_/Ag	18.0	0.80	73	10.6
